# Crosstalk between endoplasmic reticulum stress response and autophagy in human diseases

**DOI:** 10.1080/19768354.2023.2181217

**Published:** 2023-02-23

**Authors:** Junhee Kwon, Jihyun Kim, Keun Il Kim

**Affiliations:** Department of Biological Sciences, Sookmyung Women’s University, Seoul, Republic of Korea

**Keywords:** Endoplasmic reticulum (ER) stress, unfolded protein response (UPR), autophagy, human diseases

## Abstract

Cells activate protective mechanisms to overcome stressful conditions that threaten cellular homeostasis, including imbalances in calcium, redox, and nutrient levels. Endoplasmic reticulum (ER) stress activates an intracellular signaling pathway, known as the unfolded protein response (UPR), to mitigate such circumstances and protect cells. Although ER stress is sometimes a negative regulator of autophagy, UPR induced by ER stress typically activates autophagy, a self-degradative pathway that further supports its cytoprotective role. Sustained activation of ER stress and autophagy is known to trigger cell death and is considered a therapeutic target for certain diseases. However, ER stress-induced autophagy can also lead to treatment resistance in cancer and exacerbation of certain diseases. Since the ER stress response and autophagy affect each other, and the degree of their activation is closely related to various diseases, understanding their relationship is very important. In this review, we summarize the current understanding of two fundamental cellular stress responses, the ER stress response and autophagy, and their crosstalk under pathological conditions to help develop therapies for inflammatory diseases, neurodegenerative disorders, and cancer.

## Introduction

The endoplasmic reticulum (ER) is a continuous stack of flattened sac-shaped organelles in the cytoplasm of eukaryotic cells. It is important for the assembly of newly synthesized proteins as it mediates their folding and transport to the Golgi apparatus for further de novo protein formation (Kleizen and Braakman 2004). Most secreted proteins, transmembrane proteins, and lipids are produced in the ER. In addition, the ER maintains cellular homeostasis by tightly regulating calcium dynamics, phospholipid biogenesis, and various intracellular signals by releasing Ca^2+^ (Chevet et al. 2001; Berridge 2002; Park et al. 2021). Alterations in calcium levels and metabolism, redox imbalances, nutrient deprivation, and hypoxia can disrupt ER homeostasis (Schonthal 2012). Loss of ER capacity, termed ‘ER stress,’ results in the accumulation of misfolded and unfolded proteins in the ER due to a lack of molecular chaperones and calcium, disruption of the redox state, protein mutations, and decreased disulfide bonds (Eizirik et al. 2008). Thus, prolonged ER stress can cause serious diseases including cardiovascular and neurodegenerative diseases, diabetes, and cancer (Lin et al. 2019; Chipurupalli et al. 2021). Fortunately, eukaryotic cells respond rapidly to ER stress by activating a cellular adaptive pathway called the unfolded protein response (UPR) to relieve the ER burden, restore homeostasis, and avoid cell death (Eizirik et al. 2008; Lin et al. 2019).

In addition to the UPR, cells maintain homeostasis by regulating protein synthesis or degradation in response to environmental conditions (Wang and Klionsky 2003). Autophagy is a major intracellular degradation process in which unnecessary or dysfunctional proteins, damaged organelles, and intracellular pathogens are transported to the lysosomes for degradation (Mizushima and Komatsu 2011; Zhao et al. 2022). The major role of autophagy is to balance energy sources during development and nutritional stress (Glick et al. 2010). It also plays a role in quality control at the basal level by providing amino acids, glucose, and fatty acids derived from degraded materials (Nakatogawa et al. 2009; Mizushima and Komatsu 2011). Autophagy in mammals can be broadly classified into three types. The first is macroautophagy, a mechanism that uses a double-membrane vesicular organelle called the autophagosome (Glick et al. 2010). The phagophore, which sequesters cytoplasmic materials, expands and closes to form a complete vesicle called the autophagosome. Autolysosomes, formed by the fusion of autophagosomes and lysosomes, degrade internal substances via lysosomal hydrolases (Chen and Klionsky 2011; Mizushima and Komatsu 2011). The second type, microautophagy, induces lysosomal membrane invagination such that cytoplasmic components directly enter the lysosome (Mizushima and Komatsu 2011). The third chaperone-mediated autophagy (CMA) is autophagy in which target proteins are translocated directly across the lysosomal membrane, which is not mediated by the autophagosome and not related to membrane reorganization (Glick et al. 2010; Mizushima et al. 2011; Mizushima and Komatsu 2011). A group of chaperones, including heat shock protein 70 (HSC70), recognizes substrates with a KFERQ sequence known as the CMA targeting motif and binds to the lysosomal membrane receptor LAMP-2A to translocate the substrate (Orenstein and Cuervo 2010). Since macroautophagy is the main type of autophagy and is simply referred to as autophagy, autophagy hereafter refers to macroautophagy. In this review, we summarize the signaling and working mechanisms of two representative stress response pathways in cells: the ER stress response and autophagy. We also summarized and discussed their crosstalk at the signal level and in the context of human diseases.

## ER stress response signaling

**Figure 1.**
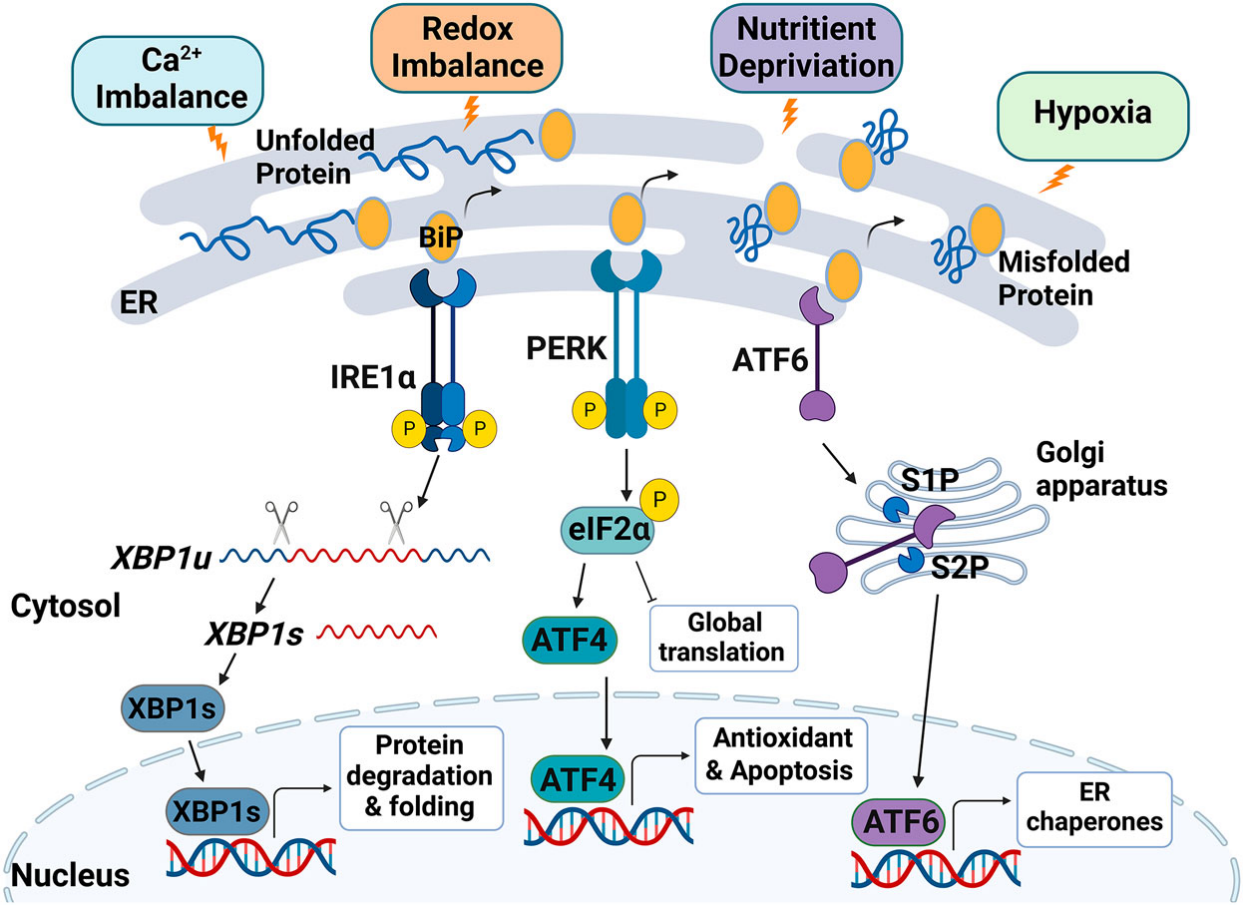
Endoplasmic Reticulum (ER) stress induces unfolded protein response (UPR) as a recovery mechanism in cells. When cells encounter stressful environments, such as imbalanced calcium and redox levels, nutrition deprivation, and hypoxia, they disrupt ER homeostasis and accumulate misfolded proteins in the ER. To relieve ER burden, the UPR is initiated by BiP dissociation from three ER transmembrane sensors: inositol-requiring protein-1α (IRE1α), protein kinase RNA (PKR)-like ER kinase (PERK), and activating transcription factor 6 (ATF6). Consequently, IRE1α oligomerizes and autophosphorylates in the ER lumen and splices transcription factor X-box-binding protein 1 (XBP1) mRNA (*XBP1u*) to produce short form of mRNA, *XBP1s*. The active form of XBP1 (XBP1s) translocates to the nucleus and upregulates the expression of genes involved in protein degradation and folding. PERK also dimerizes and autophosphorylates and then phosphorylates the translation initiation factor eIF2α to attenuate global translation. Separately, phosphorylated eIF2α increases the translation of ATF4 mRNA, allowing ATF4 to induce the expression of genes involved in antioxidant responses and apoptosis. Finally, ATF6 translocates to the Golgi compartment and is cleaved by Site-1 protease (S1P) and Site-2 protease (S2P). Cleaved ATF6 translocates into the nucleus and initiates the expression of ER chaperones and various ER stress response-associated genes, including *XBP1*.

The UPR is mediated via three ER transmembrane sensors: inositol-requiring protein-1α (IRE1α), protein kinase RNA (PKR)-like ER kinase (PERK), and activating transcription factor 6 (ATF6) (Figure 1). Under normal conditions, the binding immunoglobulin protein (BiP; also known as Grp78) binds to these three protein sensors, rendering them inactive (Munro and Pelham 1986). However, when unfolded or misfolded proteins accumulate, the major ER chaperone BiP dissociates from these sensors and binds to inappropriately folded proteins, and the sensors dissociated from BiP become active and initiate UPR signal transduction (Bertolotti et al. 2000; Shen et al. 2002). As a Ser/Thr protein kinase and endoribonuclease, IRE1α oligomerizes in the ER lumen, and its cytosolic domain is autophosphorylated once activated. It then splices the transcription factor X-box-binding protein 1 (*XBP1*) mRNA, named *XBPu*, to obtain the active form of XBP1 (XBP1s) through its C-terminal kinase and endoribonuclease domain (Flamment et al. 2012; Wu et al. 2015; Song et al. 2018; Zhou et al. 2021). XBP1s upregulates the transcription of genes that mediate protein degradation and folding. PERK, which dimerizes and autophosphorylates upon activation, phosphorylates eIF2α, a translation initiation factor that attenuates the global translation (Teske et al. 2011). In addition, p-eIF2α induces the translation of ATF4 mRNA, a transcriptional activator of genes involved in antioxidant responses and apoptosis (Teske et al. 2011). ATF6, a transcription factor, is translocated to the Golgi compartment and is cleaved by Site-1 protease (S1P) and Site-2 protease (S2P). A cleaved form of ATF6 then translocates to the nucleus and activates the transcription of ER stress response genes, such as *XBP1*, to relieve the ER burden (Song et al. 2018).

## Autophagy process and signaling

**Figure 2.**
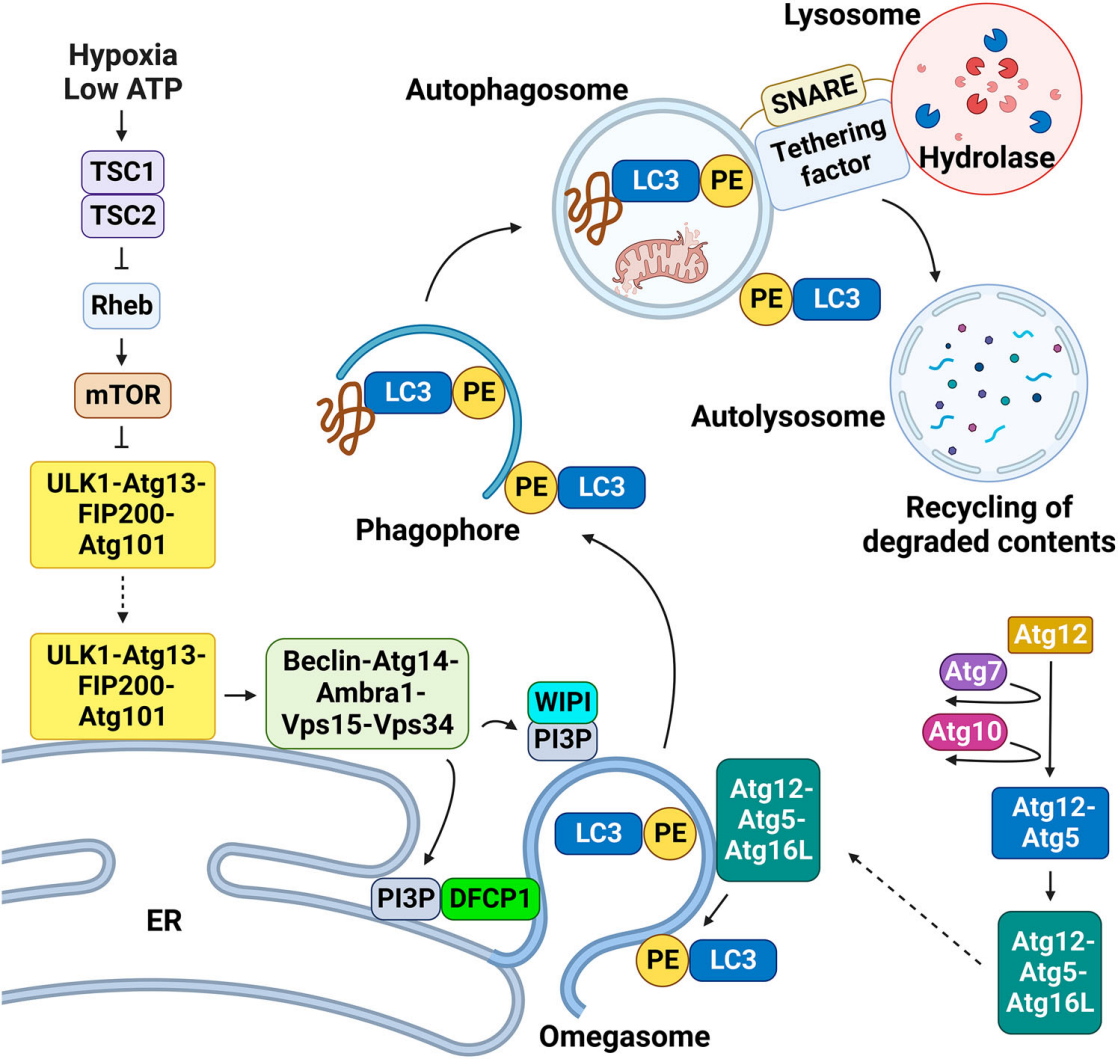
Mechanism of autophagy. Upon the induction of autophagy, the ULK1-Atg13-FIP200-Atg101 complex is activated and translocated to a specific ER domain. In the ER, the ULK1 complex activates the Beclin-Atg14-Ambra1-Vps15-Vps34 complex, and Vps34 of the phosphoinositide 3-kinase complex produces phosphatidylinositol 3-phosphate (PI3P). PI3P is recruited to double FYVE-containing protein 1 (DFCP1) and facilitates the formation of omegasomes, an ER site where a phagophore is formed. Another PI3P binding protein, the WD-repeat protein that interacts with phosphoinositide (WIPI), is also required for phagophore elongation. The Atg12-Atg5-Atg16L complex, which plays a key role in the elongation and closure of phagophores, is essential for the formation of covalent bonds between LC3 and PE. Atg5-Atg12 conjugation and LC3 processing are ubiquitin-like systems. Atg12 is activated by Atg7, which acts similarly to the E1 ubiquitin-activating enzyme. Subsequently, Atg12 is transferred to the E2-like protein Atg10 and covalently binds to the 130th lysine of Atg5. Atg12-Atg5 complex interacts with the Atg16L dimer to extend the phagophore. The site of LC3-PE conjugation was determined by the Atg12-Atg5-Atg16L complex, which serves as a specific E3-like ligase. The LC3-PE complex acts as a phagophore receptor and participates in its selective uptake by interacting with adapter molecules. Two types of complexes (STX17-SNAP29-VAMP7/VAMP812,13 and STX7-SNAP29-YKT6), which contain a protein called soluble N-ethylmaleimide-sensitive factor attachment protein receptor (SNARE), promote the fusion of autophagosomes and lysosomes. Autophagosomes that fuse with lysosomes become autolysosomes, which degrade internal materials.

Various autophagy-related (Atg) proteins play important roles in autophagosome formation (Figure 2). In the autophagy pathway, the ULK1-Atg13-FIP200-Atg101 complex activates the Beclin-Atg14-Ambra1-Vps15-Vps34 complex, and Vps34, a class III phosphoinositide 3-kinase, produces phosphatidylinositol 3-phosphate (PI3P) (Chang 2020). PI3P facilitates the formation and elongation of phagophores. The Atg12-Atg5-Atg16L complex and microtubule-associated protein 1 light chain 3 (LC3) – phosphatidylethanolamine (PE) complex play key roles in the elongation and closure of the phagophore (Glick et al. 2010; Mizushima et al. 2011; Chang 2020). Eventually, autophagosomes fuse with lysosomes to form autolysosomes that degrade internal substances (Glick et al. 2010).

The mammalian target of rapamycin complex 1 (mTORC1) prevents autophagy by inhibiting ULK1 complex formation via direct phosphorylation of Ser757 of ULK1 under nutrient-rich or growth-promoting conditions (Chang 2020). In contrast, in the absence of nutrients or hypoxia, mTOR is inhibited, and autophagy is activated (Chang 2020). Low adenosine triphosphate (ATP) levels activate adenosine 5′-monophosphate-activated protein kinase (AMPK), which, in turn, mediates the activation of tuberous sclerosis proteins 1 and 2 (TSC1/TSC2) (Saucedo et al. 2003). TSC1/2 promotes autophagy by regulating Ras homolog enriched in the brain (RHEB), which is required for mTOR activity (Saucedo et al. 2003). Moreover, AMPK directly inhibits the regulatory-associated protein mTOR (Raptor), a subunit of mTORC1, or directly activates the ULK1 complex (Chang 2020). Under conditions involving hypoxia, autophagy is initiated via mTOR inhibition by the regulation of development and DNA damage response 1 (REDD1), a major protein of the hypoxia-inducible factor 1 subunit alpha (HIF1α)-independent pathway (Brugarolas et al. 2004). In addition to mTORC1, sex steroids and their receptors have been implicated in the transcriptional regulation of autophagy-associated proteins, such as the ULK1 and PI3K complexes (Shang et al. 2021). Histone modifiers are also involved in the regulation of the transcription of core autophagy genes. For instance, H3R17 dimethylation by coactivator-associated arginine methyltransferase 1 (CARM1), or H4K16 acetylation by hMOF acetyltransferase, induces transcription of autophagy-related genes (Shin et al. 2016). Conversely, suppression of autophagy-related gene expression is mediated via H3K9 dimethylation by G9a methyltransferase and H3K27 trimethylation by enhancer of zeste homolog 2 (EZH2) (Baek and Kim 2017).

## Crosstalk between ER stress and Autophagy

**Figure 3.**
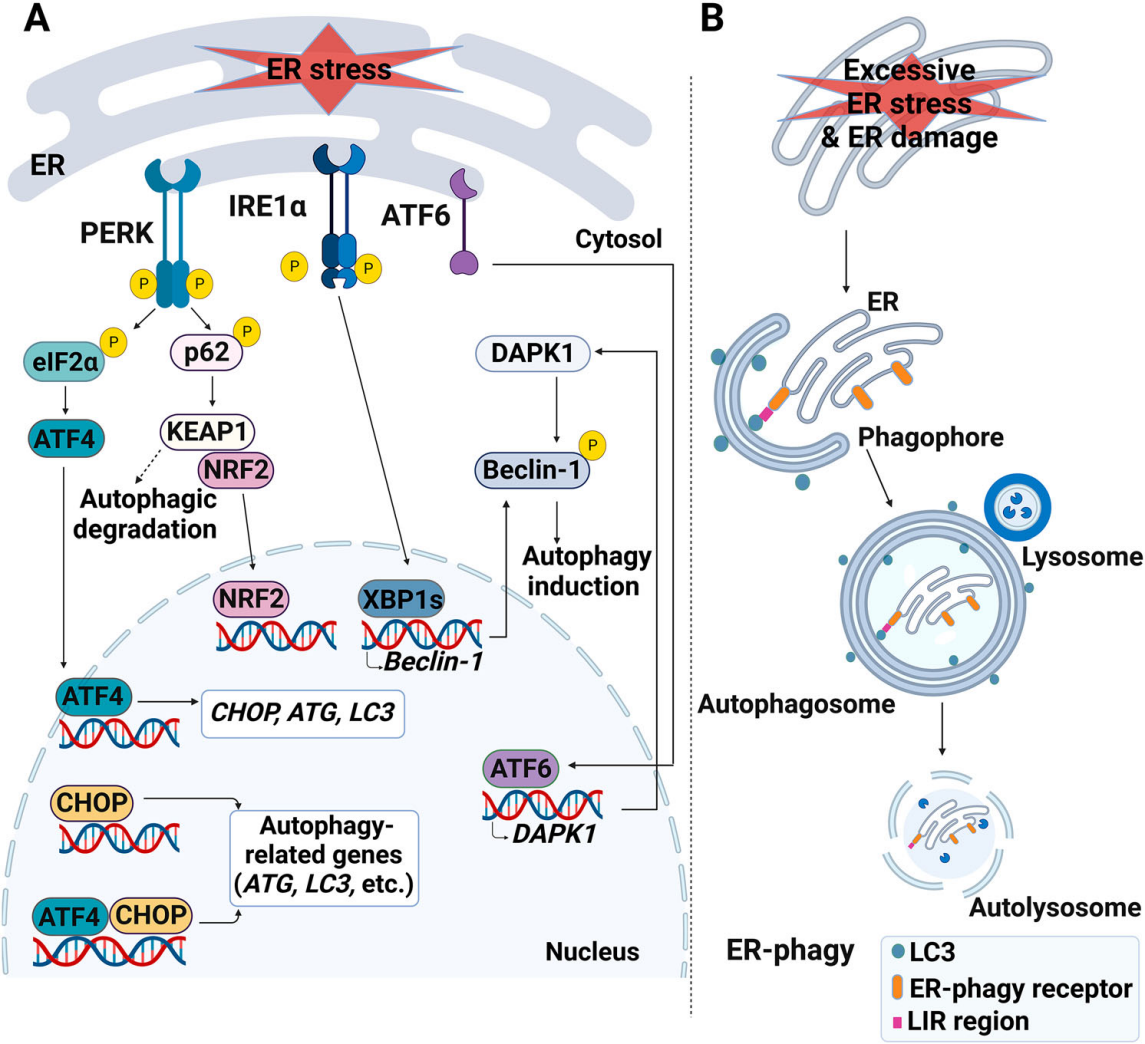
The role of the ER stress response as a potential trigger for autophagy. (A) All three UPR signals mediated via PERK, IRE1α, and ATF6 are involved in autophagy. The PERK-eIF2α-ATF4 axis, together with the transcription factor CHOP, upregulates most autophagy genes (for instance, *ATG* and LC3). CHOP is upregulated by ATF4 and participates in the expression of autophagy-related genes. In addition, PERK phosphorylates p62, which mediates the autophagic degradation of KEAP1 and nuclear translocation of NRF2. IRE1α splices XBP1 (XBP1s), which binds to the Beclin-1 promoter and upregulates its transcription, thereby promoting autophagy. Lastly, ATF6 upregulates DAPK1 expression, which, in turn, phosphorylates Beclin-1 to induce autophagy. (B) ER-phagy is initiated to remove damaged ER organelles. ER-phagy receptors on ER membrane mediate the interaction between LC3 and its target ER sites via the LC3-interacting region (LIR).

Many ER stress inducers, such as tunicamycin, arachidonic acid, DFS, and Kazinol C, are known to simultaneously induce autophagy (Kwon et al. 2020; Lee et al. 2022). ER stress and autophagy are two major response pathways to cellular stress that are closely related to each other. (Senft and Ronai 2015; Smith and Wilkinson 2017; Bhardwaj et al. 2020). UPR is one of the factors responsible for the induction of autophagy, in addition to nutrient deprivation and mTOR inhibition (Mizushima and Komatsu 2011; Deegan et al. 2013). Three UPR sensors, PERK, IRE1α, and ATF6, are all related to autophagy (Figure 3A). When ER stress is induced, the PERK-eIF2α-ATF4 signal accelerates autophagy by transcriptionally upregulating most autophagy genes (B'Chir et al. 2013). ATF4 upregulates the expression of CHOP, a transcription factor, and ATF4, CHOP, and ATF4-CHOP heterodimers enhance the transcription of autophagy-related genes (B'Chir et al. 2013). In addition, PERK-mediated phosphorylation of p62, an autophagy cargo receptor, accelerates the autophagic degradation of KEAP1, resulting in the activation of the master antioxidant transcription factor NRF2 (Ichimura et al. 2013). The IRE1α-XBP1 axis triggers autophagy by inducing the expression of Beclin-1 in endothelial cells (Margariti et al. 2013). Spliced XBP1 binds specifically to the Beclin-1 promoter and induces its transcription, leading to the upregulation of autophagy (Margariti et al. 2013). Likewise, ATF6 upregulates the expression of death-associated protein kinase 1 (DAPK1) (Gade et al. 2012), which phosphorylates Beclin-1 and mediates autophagy initiation (Zalckvar et al. 2009). Although prolonged activation of both ER stress and autophagy tends to induce cell death, ER stress-linked autophagy plays a role in promoting cell survival in most tumor microenvironments, highlighting the importance of the interplay between ER stress and autophagy in the cancer treatment (Senft and Ronai 2015).

ER stress-associated autophagy is essential for maintaining stabilized ER function via the degradation of aberrant and surplus components of the ER, as well as the damaged ER organelle itself, termed ER-phagy (Mochida and Nakatogawa 2022). ER-phagy targets the ER for degradation via fusion with the autophagosome when the ER is damaged or the acute response to the UPR exceeds the limit (Figure 3B). Certain ER-phagy receptors, such as FAM134B, SEC62, RTN3L, TEX264, ATL3, and CCPG1, are located on the ER membrane and mediate the interaction between LC3 and its target ER sites via the LC3-interacting region (LIR) (Yang et al. 2021). In addition to preventing ER dysfunction, ER-phagy plays a key role in protecting cells from damage and death attributed to robust ER stress (Grumati et al. 2018). As maintenance of ER homeostasis is vital, proper regulation of ER-phagy is also important, as otherwise, various diseases may be developed.

ER stress does not always positively regulate autophagy. In a few types of pathological conditions, especially neurodegenerative diseases, abnormal ER stress blocks autophagy (Rashid et al. 2015). Downregulation of the IRE1α-XBP1 axis in Huntington’s disease and amyotrophic lateral sclerosis induces autophagy by upregulating the forkhead box (FOXO), which is known to elevate key autophagy-related genes such as *ATG12, BECN1, BNIP3, GABARAPL1*, and *LC3,* and promote pathological conditions (Salih and Brunet 2008; Vidal et al. 2012). Although the ER stress response and autophagy act as two independent pathways, ER stress can play dual roles in initiating or inhibiting autophagy. These two systems are determinants of cell fate, and therefore, require tight control.

## Overlapping role of ER stress signaling and autophagy in diseases

### Inflammatory disease

Intestinal epithelial cells (IECs) frequently demonstrate ER stress-induced inflammatory responses due to environmental exposure to microbiota, metabolites, and toxins (Chipurupalli et al. 2021). Crohn's disease-like inflammatory diseases are induced when key regulators of the UPR and autophagy, such as XBP1 and ATG16L, are disrupted, suggesting the importance of the two pathways in regulating the inflammatory response in IECs (Senft and Ronai 2015). A group of patients with Crohn's disease, whose autophagosome formation was disrupted by the ATG16L mutation, showed frequent ER stress in Paneth cells (Adolph et al. 2013). Like the intestines, the lungs are damaged by various toxins, causing inflammatory diseases such as acute lung injury (ALI) and chronic obstructive pulmonary disease (COPD). In a study on ALI mediated by lipopolysaccharide (LPS; a component of the gram-negative bacterial membrane, which is a representative cause of lung damage), ER stress was confirmed to induce autophagy, and the autophagy inhibitor, 3-Methyladenine, increased ER stress and decreased cell viability (Zeng et al. 2017). Therefore, ER stress attributed to LPS appears to induce autophagy as a cell-protective mechanism, thereby reducing UPR, inflammatory responses, and cell death (Zeng et al. 2017). CMA alleviates UPR and apoptosis in cigarette smoke-induced COPD (Chipurupalli et al. 2021). In many cases, autophagy inhibits disease progression. However, it exacerbates the autoimmune disease multiple sclerosis. Increase in the autophagy mediated by the UPR contributes to the survival and proliferation of T cells, which are key drivers of the inflammatory response, leading to increased demyelination and neuroinflammation, which are hallmarks of multiple sclerosis (Andhavarapu et al. 2019).

### Neurodegenerative disorder (NDD)

The brain is sensitive to various stressors. When metabolism, neurotransmitters, and active metal ions increase owing to genetic and environmental factors, neurons are damaged, and NDDs are developed (Esmaeili et al. 2022). The link between the ER stress response and autophagy in Parkinson's disease (PD) has been confirmed in studies examining the effects of therapeutics on the loss of dopaminergic (DA) neurons. One study found that mesencephalic astrocyte-derived neurotrophic factor (MANF), which is effective in treating PD, activates the autophagy pathway (Zhang et al. 2018). Autophagy is activated to reduce the accumulation of α-synuclein, which is one of the causes of PD, reducing ER stress and the death of DA neurons (Zhang et al. 2018). Furthermore, small doses of morphine may activate autophagy and act as a neuroprotector via attenuation of ER stress, similar to MANF in 6-hydroxydopamine (6-OHDA)-induced PD (Wang et al. 2018). Amyotrophic lateral sclerosis (ALS) is an NDD caused by mutations in various genes involved in autophagy dysfunction (Evans and Holzbaur 2019). Although it is not a common mutation, a two-base pair insertion in the exon 6 of the *OPTN* gene is associated with ALS. In NSC-34 cells harboring this mutation (a cellular model of ALS), the sustained activity of TANK-binding kinase 1 is induced, and the formation of LC3-positive aggregates is increased (Medchalmi et al. 2021). Excessive formation of LC3-positive aggregates mediates ER stress and eventually induces apoptosis. Another ALS-associated mutation, the TAR DNA binding protein 43 mutation (TDP-43A315 T), was found to increase ER stress markers including CHOP, GRP78, and eIF2α, as well as autophagy markers such as Beclin-1 and LC3-PE (Medchalmi et al. 2021). Excessive neuronal damage occurs when cells with this mutation are treated with 3-MA, indicating that autophagy acts as a protective mechanism to reduce neuronal toxicity (Wang et al. 2015). Alzheimer's disease is caused by the abnormal accumulation of proteins, such as amyloid-β (Aβ) and neurofibrillary tangles (NFTs) in neuronal cells (Esmaeili et al. 2022). The UPR, induced by abnormal protein accumulation in Alzheimer's disease, activates autophagy but not the proteasome to degrade these aggregates (Nijholt et al. 2011). Recently, detailed mechanisms have been studied, such as a study in which ATF6 regulates cystathionine to activate autophagy and restore the memory (Zhang et al. 2022).

### Cancer

As mentioned above, long-term autophagy triggered by ER stress induces cell death but essentially plays a role in cell survival in the tumor microenvironment (Zhao et al. 2013; Lin et al. 2019). The UPR and autophagy play dynamic roles throughout melanoma progression and are abnormally increased during the metastatic stage. Many melanomas carry gain-of-function mutations in the v-RAF murine sarcoma viral oncogene homolog B1 (BRAF), which functions in cell division and differentiation and is treatable with a BRAF inhibitor (BRAFi) in these cases (Meng et al. 2015). However, most patients with melanoma who are treated with BRAFi develop resistance associated with ER stress-induced autophagy. Therefore, autophagy inhibitors are effective in treating BRAFi-resistant melanoma (Ma et al. 2014). In osteosarcoma cells, PERK, which is involved in UPR signaling, induces autophagy by inhibiting mTOR, an autophagy suppressor (Ji et al. 2015). As autophagy prevents apoptosis of osteosarcoma cells, osteosarcoma growth may be limited by the knockdown of PERK (Ji et al. 2015). Similarly, the anticancer effect of lexibulin on osteosarcoma is also limited by ER stress-induced autophagy, and its effect on cancer cell death is more potent when used together with an autophagy inhibitor (Wang et al. 2019). Additionally, research on substances with various anticancer effects has demonstrated that ER stress induction and autophagy inhibition play an important role in the death of various cancer cells, such as liver, colorectal, and prostate cancer (Kwon et al. 2020; Lee et al. 2022).

## Concluding remarks

**Figure 4.**
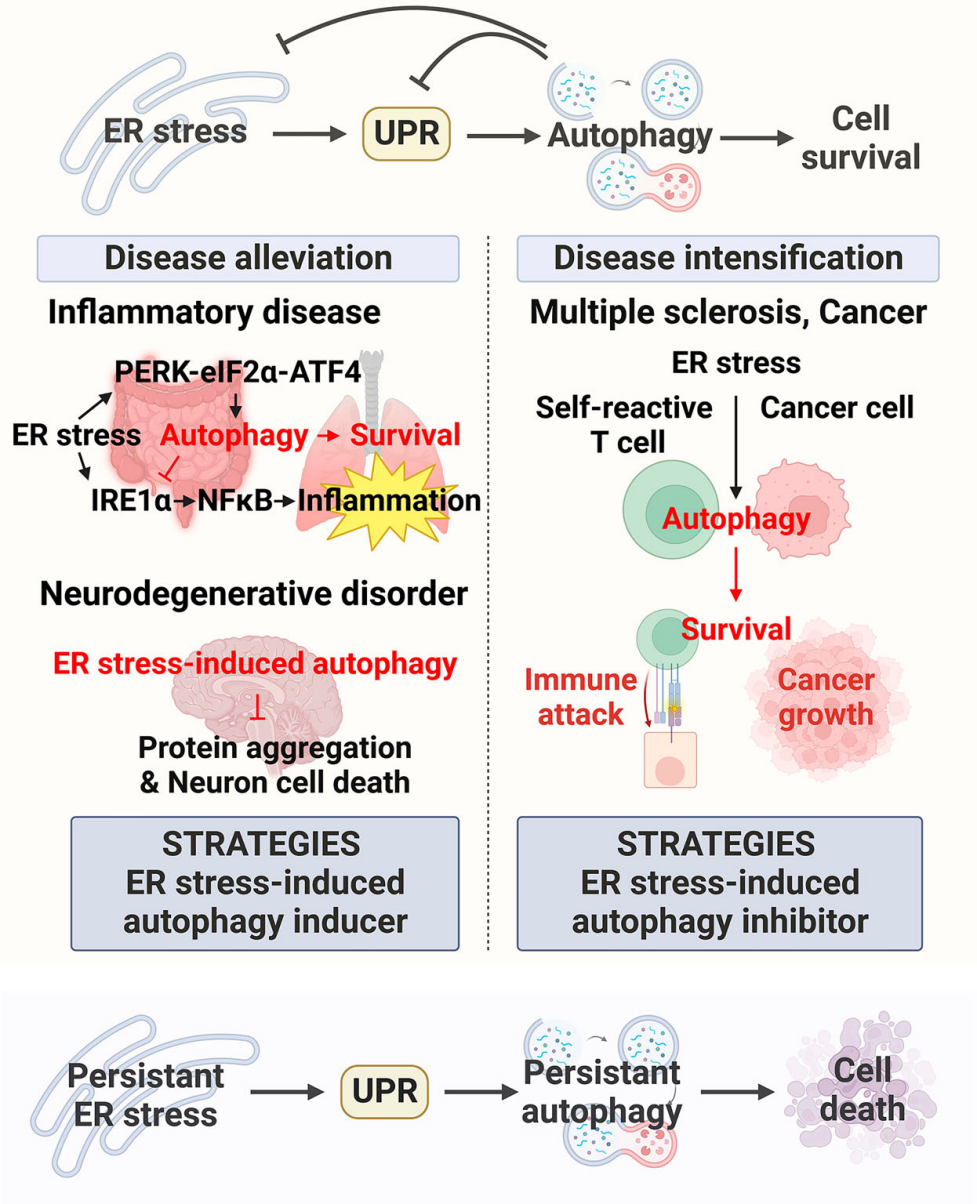
Dual role of ER stress-induced autophagy in human diseases. Properly regulated autophagy and ER stress are important for cell survival; however, excessive ER stress and sustained autophagy lead to cell death. Cells exposed to ER stress induce UPR and autophagy to minimize damage. When autophagy is disrupted in intestinal cells, Crohn's disease is induced due to excessive ER stress, or lung cells are damaged, inhibition of inflammation and cell survival through autophagy inducers alleviates the diseased conditions. In neurodegenerative disorders, treatment through ER stress-induced autophagy prevents disease progression by alleviating protein aggregation and cell death in dopaminergic and motor neurons. In contrast, in multiple sclerosis, an autoimmune disease, ER stress-induced autophagy exacerbates the disease because it augments the survival of auto-reactive T cells that cause the disease. In cancer, these survival strategies are sometimes used to maintain survival or develop resistance to anticancer drugs.

The ER stress response and autophagy, which are the representative cellular stress response pathways, work in tandem. Therefore, understanding the precise mechanism underlying the crosstalk between the ER stress response and autophagy will be of great help in elucidating pathways involved in maintaining cellular homeostasis. Although ER stress-induced UPR inhibits autophagy under some pathological conditions, accumulating evidence indicates that autophagy is activated in many cases (Figure 4). Sustained ER stress-induced autophagy tends to induce apoptosis by destroying intracellular structures. However, in most cases, autophagy promotes cell survival (Zhao et al. 2013). The roles of these two systems in determining cell fate provide important directions for elucidating the causes and treatments of various diseases. In inflammatory diseases or neurodegenerative disorders, ER stress occurs in certain cells and promotes cell death. To prevent this, the activity of the UPR, which regulates ER stress and autophagy, is increased in cells because autophagy plays a role in maintaining cell survival. In this context, several therapeutic studies in NDD have sought to exploit mechanisms that further increase ER stress-associated autophagic activity. Conversely, in autoimmune diseases or cancers, the disease is exacerbated by the use of autophagy by T cells or cancer cells to withstand ER stress. Therefore, many attempts have been made to achieve anti-cancer effects by inhibiting autophagy while stimulating ER stress. In addition, since autophagy-related genes are difficult to regulate, new anticancer approaches targeting the UPR or non-canonical ER stress are emerging by targeting known upstream signals of autophagy (Alam et al. 2022).
